# Enhancing Career Decision Status of Socioeconomically Disadvantaged Students Through Learning Engagement: Perspective of SOR Model

**DOI:** 10.3389/fpsyg.2022.778928

**Published:** 2022-09-15

**Authors:** Michael Yao-Ping Peng, Xiaoyao Yue

**Affiliations:** ^1^School of Economics and Trade, Fujian Jiangxia University, Fuzhou, China; ^2^Business School, Foshan University, Foshan, China; ^3^College of Teacher Education, Yuxi Normal University, Yuxi, China

**Keywords:** career decision status, deep approach to learning, employability, problem-based learning, self-efficacy, socioeconomically disadvantaged undergraduate

## Abstract

Higher education plays the role of cultivating talents in national development and meets the talent sources needed by the development of the state, industries and enterprises. Besides, for students, higher education can provide stimuli to improve the development of family and personal career. Especially for socioeconomically disadvantaged Students, higher education means the main factor for turning over the Socio- Economic Status. Universities endow students with abundant employment skills, so as to make them more confident in contending with the challenges in the job market. However, innate pessimism or negative attitudes and cognition may exist in socioeconomically disadvantaged Students, thereby providing effective learning context to improve their learning engagement. This study explores the influence on students’ career decision status from deep approach to learning, problem-based learning, self-efficacy and employability. A total of 627 valid questionnaires are collected in this study. PLS-SEM was adopted to verify the structural relationship in data analysis *via* SmartPLS. The results indicate that deep approach to learning and problem-based learning have significant impacts on students’ self-efficacy and employability; self-efficacy has significant impacts on employability and career decision status; employability has significant impact on career decision status; and that self-efficacy and employability play significant mediating roles in the research framework.

## Introduction

Career decision status is one of the most complex and important decisions an individual faces in life (Bimrose and Mulvey, 2015). They have a profound impact on an individual’s economic, social and mental health. However, planning a future career path can be a difficult process (Gati and Levin, 2014; Lipshits-Braziler et al., 2015; Levin et al., 2020). The reality is that many college students generally find it difficult to decide their challenging in terms of major and/or potential career path. Some of them have a harder time making career decisions after completing their degree. This career indecision can negatively impact students’ social, personal and professional lives. The reality is that many college students generally find it challenging to decide on their major and/or potential career path. Some of them have a harder time making career decisions after completing their degree. This career indecision can negatively impact students’ social, personal, and professional lives (Lam and Santos, 2018; Chuang et al., 2020). Career decision status may relate to the choice of occupation and the education and training involved, whether to continue in one job or change to another, what formal and informal advanced training to attend, etc. When faced with such decisions, the difficulty that many people encounter is often preventing them or leading to suboptimal choices (Kulcsa et al., 2020).

Although the influence of Socio-Economic Status (SES) on the growth of individuals has been extensively taught by social education (Perry and McConney, 2013; Castillo-vergara et al., 2018). However, in the past, it was mainly concentrated on the SES disadvantages of the younger growth stage, as well as the family environmental resources restrict the learning status and performance at school (Destin et al., 2019), such as: dropout tendency (Perry and McConney, 2013; Shogren et al., 2018). Regardless of the theory or practice of higher education, it is very important to explore the career-decision making of college students who grew up in SES families. (Jenkins et al., 2013; Hsieh and Huang, 2014; Destin et al., 2019). In terms of career development prospects, with the socioeconomically disadvantaged brought about by the unequal economic resources of the family, the poor living environment and physical health, lack of social networks that contribute to academic or career development (Perry and McConney, 2013; Shogren et al., 2018), as well as being affected by long-term deprivation of educational opportunities and insufficient skills, may potentially cause social exclusion, directly reflect the main development tasks that must be faced at this stage, such as Career choice and career decision (Hsieh and Huang, 2014). However, few studies have focused on socioeconomically disadvantaged students who are about to enter the workplace in the early stage of adulthood (Jenkins et al., 2013; Hannon et al., 2017), or focus on exploring the academic transition process and career decision status issues at this stage (Hsieh and Huang, 2014). In addition, some scholars have identified socioeconomic status as a factor affecting student outcomes and careers, for instance, investigated the effect of socioeconomic status (SES) on students’ math achievement growth (Langenkamp and Carbonaro, 2018); or the socioeconomic status (SES) as a unique factor influencing college students’ career decision self-efficacy (Shin and Lee, 2018); socioeconomic Status is the most important predictor of career aspirations in STEM (Mau and Li, 2018; Tran et al., 2020); the positive effects of socioeconomic status and CSCC (career success criteria clarity) on CDSE (career decision-making self-efficacy) (Xin et al., 2020), and the relationship between students’ low socioeconomic status (SES) and employment aspirations (Gore et al., 2015; Saw et al., 2018). Therefore, this study attempts to explore the issues related to factors that affect the career choices of socioeconomically disadvantaged students. However, in these studies, there is no exploration from the perspective of SOR, Career Decision Status and Socioeconomically Disadvantaged Students are only a variable in the model, and the reasons that affect career choice are not specifically explored. And there is no study that uses self-efficacy and employability as mediating variables. Therefore, the innovations of this paper are as follows: 1) Based on the background of Career Decision Status and Socioeconomically Disadvantaged Students, explore factors that affect career choice; 2) From the perspective of SOR theory, find learning strategies that are conducive to improving career choice; 3) Use self-efficacy and employability as mediating variables.

Factors such as the learning status and learning input in the school are more likely to affect the status acquisition and the quality of employment life after entering the workplace (Hannon et al., 2017), and restrict long-term career development (Hsieh and Huang, 2014), and the career adaptability that can be used in the future to continuously respond to career challenges in the work. Socioeconomically disadvantaged students have long-standing low self-esteem, lack of autonomy and sense of control due to being in a disadvantaged situation (Perry and McConney, 2013; Destin et al., 2019; Perry and McConney, 2013), it may cause socioeconomically disadvantaged students to experience more psychological dilemmas such as indecision, helplessness, and lack of hope when facing career choices than their peers (Jenkins et al., 2013; Shogren et al., 2018; Destin et al., 2019; Jenkins et al., 2013; Shogren et al., 2018), as a result, there is a phenomenon of conflict or difficulty in making choices, which is manifested in trait anxiety (Kalaycioglu, 2015), or the long-term chronically indecisive affects the meaning of life (Perry and McConney, 2013). Therefore, certain external stimuli must be used to attract these socioeconomically disadvantaged students to improve their learning input (Jenkins et al., 2013). This study uses the S-O-R model, which includes three parts, namely stimulus, organism and response, to link the learning mode of socioeconomically disadvantaged students in the learning process with the career decision status. In the part of learning stimulus, many scholars emphasize that the application of learning methods can help arouse students’ learning motivation and learning gains (Baglama and Uzunboylu, 2017), such as deep approach to learning and problem-based learning, provide suitable learning methods, and provide socioeconomically disadvantaged students with strong learning stimulation in a timely manner (Miller and Rottinghaus, 2014). Therefore, this study attempts to use the SOR model to explore the stimulation process of socioeconomically disadvantaged students in career decision status.

In the SOR model, the stimulus factor adopts the deep approach to learning and problem-based learning, and the response factor adopts the career decision status of socioeconomically disadvantaged students, and the intermediate effect is that the response after the stimulus is effective in the subsequent behavior of the individual attitude or intention, such as learning initiative, learning intention (Animesh et al., 2011; Ha and Im, 2012). However, as to the changes in the internal organisms of socioeconomically disadvantaged students caused by stimulus, most studies discuss changes in attitude or psychology, but seldom discuss actual changes in cognitive behavior, especially the career decision status of socioeconomically disadvantaged students (Hsieh and Huang, 2014). Therefore, this research will propose corresponding variables from the psychological and cognitive level to organizations. In terms of the related concepts of career decision status, the most important psychological factor comes from self-efficacy, which is also the most important mediating factor in the model, which will links stimulate and reaction (Hsieh and Huang, 2014; Baglama and Uzunboylu, 2017). The self-efficacy theory points out that confidence is a state that individuals consciously want to try to achieve, and the higher the degree of self-efficacy, the more they will be able to face clear, more difficult challenges, and the more valuable they are (Hannon et al., 2017), the more it can increase their academic performance and academic achievement (Kalaycioglu, 2015), at the same time, self-efficacy can guide students to focus and work hard toward the goal, which is closely related to cognition and behavior (Kalaycioglu, 2015). At the cognitive level, the ability to improve the future career decision status of socioeconomically disadvantaged students is to improve the organisms factors of the future career decision status of students (Hannon et al., 2017), therefore, only by perceiving the improvement of employability, can socioeconomically disadvantaged students understand how to face the future career decision status, which has become the focus of this research (Hannon et al., 2017), and further explore to reduce the long-term negative impact of structural restraint and social class inequality. Such educational benefits and far-reaching effects are gradually being explored in the current career research field of socioeconomically disadvantaged students (Hsieh and Huang, 2014). Based on the above, this study intends to explore the influence of self-efficacy and employability on career decision status.

According to the research content and purpose, this research expects to propose the following theoretical and practical contributions: (1) Use the SOR Model to discuss the career decision status of Socioeconomically Disadvantaged Students; (2) Add the self-efficacy theory to combine theories and enrich the SOR model application; (3) Explore different learning modes, such as deep approach to learning and problem-based learning as the main simulates factor of socioeconomically disadvantaged students, thereby improving their subsequent career decision status.

## Literature Review and Hypotheses Development

### SOR Model in Socioeconomically Disadvantage Students

The S-O-R model is made up of three components, including stimulus, organism and response, which determine the behavioral outcome of the event. In regard to the concept of stimulus and response, it is viewed as “a part of behavior and environment.” Emergent environmental changes will influence the psychological and emotional capabilities of individuals, thus further facilitating behavioral changes. Stimulus, defined as “what influences the individual,” is the extrinsic force influencing the psychological state of the individual (Fu et al., 2020). An organism can be regarded as the intrinsic procedure and structure between an individual’s extrinsic stimulus and the final action, reaction or response. In terms of the intervention process and structure, perceptual, physiological, sensory and thinking activities are contained (Pandita et al., 2021). When it comes to environmental psychology, the stimulus-organism-response (SOR) model illustrates that various extrinsic elements can be adopted as stimuli (S), which in turn influence the intrinsic state (O) of individuals, and thus the individual’s behavioral response (R) (Fu et al., 2020; Zhai et al., 2020). Due to the acquired economic deprivation, socioeconomically disadvantaged students are affected and arranged by family factors in their growth and learning process (Jenkins et al., 2013), Therefore, the use of external stimulus models may explain how to improve the impact of socioeconomically disadvantaged students on career decision status (Baglama and Uzunboylu, 2017). In this study, the SOR model contributes to accounting for changes in the psychological cognition of socioeconomically disadvantages students while learning, as well as future learning intentions and behavioral responses. As for the intrinsic psychological changes derived from the individual who is being stimulated by the surroundings, the SOR model is conducive to giving an explanation (Lin et al., 2020).

In the SOR model of this study, in order to confirm whether socioeconomically disadvantaged students will be subjected to the correct learning method, it will affect the response of career decision status. Therefore, in the stimulation part, deep approach to learning and problem-based learning are used as important antecedents. However, in the organization, most studies in the past emphasized the psychological factors of inner cognition, although the stimulus can be effectively transformed into a clear response through the inner, but whether it can promote the improvement of the inner substance, there are few studies to verify it. Career decision status depends on the degree to which students have acquired self-confidence recognition and employment skills. Self-understanding can change the difficulty of career decision status. Therefore, this study uses self-efficacy and employment as the organism factors.

### Employability

With the increasing competition in the higher education (HE) sector, people are paying more and more attention to the employment results of graduates as a way to measure the quality of institutions and the return on investment of student degrees (Blackmore et al., 2016); Hillage and Pollard (1998) identified three types of “employability assets”: graduates’ knowledge (what they know), skills (what they do with what they know), and attitude (how they do it). Employability is largely regarded as a measurable economic achievement of graduates and universities. This not only highlights the importance of graduates as key contributors to economic development, but also highlights the role of higher education in promoting the training of graduates for the labor market (Fakunle and Higson, 2021). It has become a common practice to embed employability at the core of the excellent teaching framework (Department for Business, 2016). Higher Education Institutions (HEI) values employability, expects and improves students’ learning outcomes, especially at the undergraduate level.

Employability is a strategic directive of HE (Smith et al., 2018), which contains rankings of employment achievements, and government funding for higher education depends on the performance of graduates, such as the United Kingdom’s excellent teaching framework and performance-based grants from Australian universities. From the perspective of human capital, the development of skills and knowledge will increase the economic value of individuals, thereby enhancing their career prospects. The national higher education policy links outstanding professional capabilities with enhanced employment results. Whether it is right or wrong, the university’s task now is to develop these and assume the responsibility of cultivating students’ employability (Jackson and Bridgstock, 2020); Chhinzer and Russo (2018) explored employers’ perceptions of graduate employability. The results show that there is a positive relationship between professional maturity, soft skills, problem-solving ability, continuous learning and academic achievement, and employers’ perceptions of graduate employability. Employers will consider general skills (time management, teamwork, attention to detail), general psychological skills, specific subject knowledge, work willingness, work attitudes and behaviors, and the ability to respond to emergencies when assessing the employability of graduates. Frankham (2017) researched the “employability” in the Teaching Excellence Framework (TEF). First, he questioned the government’s requirements for employability and raised a series of obvious problems. Second, he reviewed recent studies on employability by other scholars and found that the goals and results of the “Employability Initiative” did not match. Third, he found that students’ attention to employment has a positive effect on employability. On the surface, students know the importance of employment, but they do not have the abilities that employers need.

The effective career choice depends on the degree of the college students’ mastery of their knowledge, skills and abilities. When students have the ability to perceive the growth of their knowledge and skills or to anticipate future career development, it is conducive to appropriate career choices (Peng, 2019). However, for poor students’ career choices, the expression and acquisition of employability becomes extra important. Lent et al. (2017) pointed out that the degree of students’ confidence in their employability is more important than their understanding of certain professional abilities, because they can moderately adjust their self-concept and mentality, and overcome learning obstacles to acquire more abilities (Creed and Gagliardi, 2015). When poor students obtain higher employability, they will be more satisfied with their future career choices and have a higher sense of identity (Burton and Beccaria, 2013). Similarly, many studies have pointed out that employability will have a greater influence on career choices than students’ professional knowledge and skills. Therefore, we put forward the following hypothesis:

H1: Student employability has a positive impact on career decision.

### Self-Efficacy

Social cognition scholars believe that under certain circumstances, an individual’s behavioral results will be affected by environmental and cognitive factors (Van Dinther et al., 2011), especially those beliefs that lead to success and behavior. They regard these beliefs as self-efficacy. It is an important cognitive variable that explains how individuals form behaviors and interact with the environment (Komarraju and Nadler, 2013; Lent et al., 2014). Research on self-efficacy is important because research shows that it can be a reliable and effective predictor of performance results, including academic performance and behavior (Brown et al., 2011; Lent and Brown, 2019). The most effective way to create a strong sense of efficacy is through mastering the experience (Lent et al., 2014; Sheu et al., 2018). Successful performance enhances a person’s self-efficacy, while failure weakens it. In addition, the success of observing social patterns through continuous efforts will increase the observer’s belief that he or she can perform well in similar activities. In contrast, despite failed persevering in observing other, it will reduce a person’s efficiency. Li et al. (2020) believe that the study of the interaction between cognitive activation and self-efficacy has important practical significance. Socioeconomically disadvantaged has a negative impact on learning outcomes. In addition, there is also a correlation between learning outcomes and self-efficacy (Wiederkehr et al., 2015); Desimone and Long (2010) pointed out that it is important to strengthen education equality, explore the role of school teaching in improving the socioeconomically disadvantaged groups, and reduce the gap of self-efficacy and learning outcomes between socioeconomically advantaged and socioeconomically disadvantaged.

Bandura (1986) theorized the central role played by perceptual self-regulation. The effectiveness of one’s academic self-development and function. In his theory, self-efficacy affects a person’s motivation, which in turn leads to self-regulated learning (Lent et al., 2014, 2018; Brown and Lent, 2019). This sequence can eventually produce ideal situations with various positive results. For example, it may lead to self-monitoring of one’s own activities and cognitive and social conditions. It may also increase the ability to adopt effective strategies to achieve near-term goals. In addition, it can carry out self-influence, including self-motivation measures and social support, to maintain one’s academic pursuit (Peng et al., 2018). The numerous benefits of self-efficacy are not surprising, and then many researchers have investigated “self-regulatory efficacy,” using self-efficacy for self-regulated learning (Van Dinther et al., 2011). In the learning process, greater self-efficacy will have a positive impact on students’ learning motivation, cognitive ability, academic interest, emotional management and achievement (Peng et al., 2018). Based on the SOR model, scholars have argued that learning stimuli under different learning engagements do not necessarily generate positive learning performance and decision behaviors, because the generation of them depends on the improvement of intrinsic psychological traits and the intense belief in achieving work goals in the future (Zhai et al., 2020; Zhang et al., 2021). Learning stimuli may have a positive effect on subsequent behaviors (Zhao et al., 2021). However, the lack of multiplied strengthening at the cognition and attitude level may cause students to fail in obtaining practical skills and high confidence from their learning behaviors (Xu et al., 2021). This makes it difficult to have a good plan for career development in the future (Xin et al., 2020). Self-efficacy can also be seen for a strong and positive self-awareness, as well as the process of solving problems and completing tasks through high self-efficacy that positively affects students’ career decision status (Cacciolatti et al., 2017; Burga et al., 2020). Self -efficacy has a positive and significant impact on the career decision status of students. Based on the above, we suggest the following hypothesis:

H2: Self-efficacy plays a significantly positive impact on student career decision status.
H2a: Self-efficacy plays a mediator between deep approach to learning and career decision status.
H2b: Self-efficacy plays a mediator between problem-based learning and career decision status.

Based on the above arguments, compared with those who are not confident in capabilities, students with confidence in capabilities are accessible to more efficient behaviors and better interpersonal relationships (Brown et al., 2011; Chin and Rasdi, 2014; Chang and Edwards, 2015); Chin and Rasdi (2014) argued that students who are highly self-motivated seek professional knowledge to complete tasks in their own social networks (Lent et al., 2011; Thompson et al., 2016). Only by gaining and keeping more self-efficacy can they achieve their goals and develop higher employability (Lent et al., 2011; Jemini-Gashi et al., 2021). Furthermore, for socioeconomically disadvantage students, self-efficacy can also be seen as a strong learning attitude, and the process of students solving problems and achieving tasks through internal positive attitude will positively affect their employability (Chang and Edwards, 2015). The following hypothesis is set up:

H3: Self-efficacy plays a significantly positive impact on student employability.

### Deep Approach to Learning

Recently, DAL has received more and more attention from scholars in higher education research. Most research on DAL originated from Marton and Säljö (1976). The main argument is that students can use different methods to learn, and learning methods are closely related to learning outcomes (Ramsden, 2003; Duff and McKinstry, 2007). Deep approach to learning emphasize the purpose of understanding and applying critical thinking; surface approach to learning emphasize memory and fragmented knowledge. surface approach to learning is a learning goal achieved through rote learning (Marton and Säljö, 1976; Asikainen and Gijbels, 2017). Under DLA, students actively participate in the learning process, connect their ideas and find learning models and principles, and ensure that they understand the concepts they have learned (Bobe and Cooper, 2018). The development process of DLA is through the cooperation of students, colleges, universities and teachers to develop in-depth and specific teaching models, such as inducing students to respond positively, establishing students’ prior knowledge, and imparting more connections between thoughts (Asikainen and Gijbels, 2017). Advanced learning emphasizes that students consider their courses are favorable to bringing training for advanced thinking skills, such as analyzing the basic elements of ideas, experiences or theories, and integrating ideas, information or experience with new and more complicated explanations, and conducting their own discussion on the information value and the application of practical issues. Integrated learning contains student engagement in various fields, which integrates ideas and different opinions from various sources, such as the capability of discussing ideas and opinions with other students in academic works. The core concept of reflective learning lies in the situation that students conduct learning and expansion of their understanding by learning their own ideas, and eventually put their new knowledge into use in life (Laird et al., 2008; Pascarella et al., 2013). Based on the research purpose and research object, and referring to the research of Laird et al. (2008), this research uses advanced learning, integrated learning, and reflective learning as the measurement variables of DAL. The ability to discuss ideas and opinions. The core concept of reflective learning lies in the situation that students conduct learning and expansion of their understanding by learning their own ideas, and eventually put their new knowledge into use in life (Laird et al., 2008; Pascarella et al., 2013). In line with the purpose and object of the research, and referring to the research from Laird et al. (2008), this research uses advanced learning, integrated learning, and reflective learning as the measurement variables of DAL.

Some scholars have emphasized investigations on learning method/approach among college students (Tong and Song, 2004). Nonetheless, few studies up to now have taken advantage of these students’ learning engagement and general self-efficacy (Evans et al., 2017). In previous studies, learning modes can be divided into several ways by students, including deep learning, exploitative learning and explorative learning, etc. (Dolmans et al., 2016; Varunki et al., 2017). In different situations, the corresponding effect varies based on different learning modes. Some scholars pointed out that DAL can improve students’ learning input and enhance their learning effectiveness. Through DAL, socioeconomically disadvantaged students can reflect on their own knowledge and skills, and understand how to apply these skills in real life and solve problems (Varunki et al., 2017). In order to strengthen self-confidence of achieving learning goals and tasks, it is necessary to facilitate students engage in DAL (Komarraju and Nadler, 2013; Dolmans et al., 2016; Burga et al., 2020). Scholars suggest that the key to students gaining more confidence for problem-solving and task achieving is the need for a high degree of deep learning, thus, they can be involved in learning activities relevant to knowledge integration, reflective learning and problem-solving with consciousness. In other words, socioeconomically disadvantage students with more engagement in DAL have higher self-efficacy (Komarraju and Nadler, 2013). In summary, the study proposes the following hypothesis:

H4: Students’ deep approach to learning plays a significantly positive impact on Self-efficacy.

It is found from the study that the improvement of student employability arising from the DAL shows significant. In previous literature, the comparison between deep and surface learning was emphasized, and in recent years, there is a lack of studies on whether the DAL can promote student employability in an effective way (Cacciolatti et al., 2017; Varunki et al., 2017). Nevertheless, research findings indicate that DAL shows a positive relationship with student learning outcomes, knowledge integration, learning engagement and skills, etc. (Nelson Laird et al., 2006, Laird et al., 2008). It also presents that students are inclined to adopt DAL to acquire more explicit knowledge in the HEIs with a well-established learning assessment (Dolmans et al., 2016). Based on a succession of general knowledge of learning process, higher-order learning, integrative learning and reflective learning will make students accessible to contending with difficulties, accomplishing course tasks, acquiring new knowledge and further improving core competence (Oleson and Hora, 2014; Dolmans et al., 2016; Varunki et al., 2017). Besides, students will be enabled to focus on not only knowledge acquisition, but also the improvement of substantive learning and comprehension of their deep implications through the guidance of DAL (Laird et al., 2008; Cacciolatti et al., 2017). All the efforts contribute to improving the critical mind, skills of problem-solving and other skills of students involved in employment (Pascarella et al., 2013). In conclusion, this study suggests the following hypothesis:

H5: Deep approach to learning plays a significantly positive impact on student employability.

### Problem-Based Learning

PBL adopts the principles of constructivism to promote the application of prior knowledge, collaborative learning and active participation. When starting a PBL activity, a small group of students analyze the problem, determine the relevant facts, and apply existing knowledge and experience to solve the problem (Zhou, 2018). Compared with the problem-based learning (PBL) method, the traditional learning method seems to be related to the lower level of students’ knowledge and skills (Brinkman et al., 2021). PBL aims to simulate active learning, enabling students to work in groups and learn a topic in the context of actual problems, such as case-based discussions. In the past 30 years, people’s interest in PBL has increased, and many PBL courses have been proven to improve students’ learning abilities (Brinkman et al., 2021). PBL has experienced a renaissance, school leaders are eager to increase student participation and enthusiasm, and encourage clearer guidance on the conceptual understanding and disciplinary practice advocated by the Common Core State Standards and the next-generation scientific standards. The concept of PBL is actually popular from constructivist pedagogy. Explained by Dewey (1916), and later successfully implemented in professional training programs such as medicine, engineering, and law schools (Mergendoller et al., 2006). In problem-based and project-based learning, students learn key knowledge and improve self-awareness by solving real problems or completing projects that reflect social needs. Cutucache et al. (2016) developed a project called NE STEM 4U. In 2013, the University of Nebraska at Omaha (UNO) launched the Science, Technology, Engineering, and Mathematics 4U Program (NE STEM 4U). The program adopts the problem-based learning (PBL) teaching model to cultivate the problem-solving ability of socioeconomically disadvantaged students. The study of Secgin and Sungur (2020) examined the influence of problem-based learning (PBL) on the learning attitude of socioeconomically disadvantaged students. They used the experimental group and the control group to compare the study. The experimental group uses PBL teaching, while the control group uses traditional teaching. It turns out that students are more interested in PBL classrooms. In the PBL class, students search for information and write reports to improve their learning outcomes.

In problem-based learning, students learn basic content while solving highly complex and unclear problems, but what they learn depends on how they conceptualize the problem and propose potential solutions. PBL can increase student participation and enthusiasm (Sutton and Knuth, 2017). PBL emphasizes student-centered teaching and divides the learning process into five stages: asking questions, establish hypotheses, collect data, demonstrate hypotheses and summarize (Peng et al., 2018). In complex but meaningful problem situations, students acquire and develop the knowledge needed to solve problems through learning, and cultivate the ability of independent learning (McGrath et al., 2006). Regarding the measurement of PBL, Chang et al. (2012) proposed “problem solving” and “knowledge sharing.” Problem solving requires the use of resources to break existing thinking patterns and regroup ideas to solve problems and challenging situations. Knowledge sharing is the process of building consensus and focusing on problem solving. Through the exploration and combination of ideas, knowledge is integrated and constructed, and knowledge sharing between individuals is realized (Peng et al., 2018).

Studies have shown that learning opportunities can positively improve individual abilities and outcomes, thereby enhancing their self-efficacy (Uchida et al., 2018). In order to improve self-efficacy, students must have a long-term learning experience. The participation of students in learning challenges and the development of knowledge will increase the resources they can devote to learning challenges so as to obtain appropriate learning experiences. (Tan, 2016). Thereby, for learning activity design, in addition to internal incentive, it is necessary to encourage students to seek the meaning of learning during knowledge exploration, and shape their long-term learning objectives and personal career direction. Dunlap (2005) found that PBL makes students accessible to the acquisition of professional knowledge and skills in an effective way. However, despite such knowledge can improve learning effect, the effect may be limited if self-efficacy is not preconditioned (Peng et al., 2018). Hence, problem-oriented learning strategies should emphasize the establishment of short—and long-term objectives and provide feedback on students’ learning outcomes as a source of learning improvement, thus enhancing their sense of self-efficacy. Based on the above, the following hypothesis can be derived:

H6: PBL has a positive and significant impact on students’ self-efficacy.

Finally, as for the relationship with student employability, problem-based learning contributes to interest enhancement for students in the acquisition and utility of their professional skills, and further conducting capability improvement for students (Martin et al., 2008). It is available for students to develop attitudes toward better learning and capability of critical mind when they content with practical problems, such as critical analysis, problem resolving and reflection. According to Duncan and Al-Nakeeb (2006), students involved in problem-based learning will make changes in their learning incentives, attitudes and behaviors, so as to improve their critical mind, learning autonomy and capabilities that are related to employment. Thereby, the following hypothesis is put forward:

H7: Problem-based learning will positively correlate to student’s employability.

Based on the above hypothesis, this study proposes the following research framework:

## Methodology

### Participants and Procedure

The purpose of this study is to explore the career decision status of socioeconomically disadvantaged students in the learning process, and to analyze the impact of learning methods on self-efficacy and employability in stimulus-organism-response (SOR) model. The research sample in this study comprised socioeconomically disadvantaged students. Purposive sampling was applied. Moreover, the definition of “socioeconomically disadvantaged” is diversified in different countries. The definition from Taiwan limits it, as students in Taiwan are taken as the research object in this study. The criteria for classification of economically disadvantaged students are subject to the low-income family which is defined by the Ministry of Health and Welfare in Taiwan. The low-income family means the average monthly income per capita for the family is lower than 363.2 USD (10,869 TWD) for the absolute standard of living (Li et al., 2020). This study selected 6 Taiwanese universities, and then sent 1,000 questionnaires to them. In the questionnaire, participants were informed of the research purpose, research ethics and low risks, and the questionnaire information was processed in an anonymous way. Each questionnaire was wrapped in an envelope to ensure confidentiality and alleviate participants’ potential concern about being evaluated. After sampling, a total of 627 questionnaires were returned, for an effective response rate of 62.7%. Regarding the sample structure, 65.3% of participants were male and 34.7.2% female. Most students (84.3%) had not applied for a grant, and the study focused on respondents from the social sciences (64.3% in total).

Furthermore, the study collects information from the same respondents in form of a single questionnaire, which may lead to the common method bias (CMB). In this study, the single factor verification from Harman is adopted and all the measured items are analyzed by the non-rotating matrix. The analysis results demonstrate that there are nine factors, of which the eigenvalue is greater than 1, and the explanatory variance of factor 1 is 37.18% that could not explain most of the variance. Therefore, it can be concluded from the verification results that there is no common method bias in this study.

### Instrument

The construct of Deep approach to learning was divided into higher-order learning (HL), integrative learning (IL), and reflective learning (RL). This study adopted the scale proposed by Campbell and Cabrera (2014), Laird et al. (2008), Pascarella et al. (2013), the higher-order learning was measured using 4 items; the integrative learning was measured using 5 items; and the reflective learning was measured using 2 items, such as “How the instructional and learning environments of liberal arts colleges enhance cognitive development,” “Worked on a paper or project that required integrating ideas or information from various sources” and “Examined the strengths and weaknesses of your own views on a topic or issue.” The construct of Problem-based learning was divided into knowledge-sharing (KS) (3 items) and problem-solving (PS) (3 items), such as “Using electronic resources to support problem-based learning” and “Utilizes relevant resource materials effectively.” This study adopted the scale proposed by Chang et al. (2012).

Student self-efficacy can be defined as the degree of an students’ perceptual ability to achieve their tasks and goals. The scale was revised to integrate six items of higher reliability and validity by Rigotti et al. (2008), such as “When I am confronted with a problem in my learning tasks, I can usually find several solutions” and “Whatever comes my way in my learning tasks, I can usually handle it.” Student employability was measured using latent variables proposed by Pan and Lee (2011), which including general ability for work (GAW) (8 items), professional ability for work (PAW) (4 items), attitude at work (AW) (3 items) and career planning and confidence (CPC) (3 items), such as “Expression and communication,” “Professional knowledge and skill,” “Understanding of professional ethics,” and “Understanding and planning of individual career development.” In the “Career-decision Status” (CDS) section, this study is defined as designed for participants who needed to make a decision about their major or occupation. In this study, the career-decision proposed by Gadassi et al. (2015) were converted into a six-items scale to best describe their career-decision status, such as “I know what I will do once I graduate” and “I know what I want to do when I graduate, but I want to make sure that it is the most suitable option.” All items have been measured on a five-point Likert scale (1 = totally disagree; 5 = totally agree).

### Data Analysis Strategy

This study tested the hypotheses of the research framework and included paths *via* structural equation modeling. Firstly, in order to test the construct validity, confirmatory factor analysis (CFA) was performed using AMOS 23.0 and SPSS 23.0. Secondly, this study adopted partial least squares structural equation modeling (PLS-SEM) to build the structural model; specifically, verification of path relationship and indirect effects was performed using SmartPLS 3.0.

## Results

### Measurement

All latent variables evaluated were found to be reliable in this study, with Cronbach’s α ranging from 0.83 to 0.96. Table 1 shows the reliability of each latent variables. In order to verify validity of measurement model, this study conducted confirmatory factor analysis (CFA) *via* AMOS 23.0 to examine the construct validity, including convergent and discriminant validity. Based on validity criteria recommended from Hair et al. (2010), CFA results show that standardized factor loadings were higher than 0.5; average variance extracted (AVE) ranges between 0.602 ∼ 0.783; and composite reliability (CR) ranges between 0.907 ∼ 0.935. All three criteria for convergent validity were met, and correlation coefficients were all less than the square root of the AVE within one dimension, suggesting that each dimension in this study had good discriminant validity. Fit indices greater than 0.90 benchmark (GFI = 0.93, AGFI = 0.91, TLI = 0.97, and CFI = 0.97) indicated data fits said model. Similarly, levels of misfit were tolerable, with RMSEA = 0.058 and RMR = 0.043, which RMSEA and RMR were below the relevant benchmark of 0.08.

**TABLE 1:** Verification of measurement model.

	1	2	3	4	5	6	7	8	9	10	11
1. HL	*0.885*										
2. IL	0.802	*0.815*									
3. RL	0.747	0.744	*0.911*								
4. KS	0.578	0.647	0.553	*0.881*							
5. PS	0.650	0.607	0.611	0.690	*0.878*						
6. Self-efficacy	0.697	0.629	0.599	0.523	0.650	*0.822*					
7. GAW	0.536	0.520	0.512	0.521	0.547	0.507	*0.776*				
8. PAW	0.525	0.488	0.458	0.488	0.521	0.500	0.722	*0.853*			
9. AW	0.576	0.545	0.526	0.521	0.570	0.556	0.755	0.747	*0.847*		
10. CPC	0.539	0.544	0.491	0.495	0.533	0.560	0.657	0.640	0.741	*0.886*	
11. CDS	0.628	0.604	0.557	0.489	0.546	0.675	0.482	0.445	0.536	0.476	*0.964*
Mean	3.684	3.589	3.688	3.531	3.761	3.755	3.534	3.638	3.601	3.555	3.662
SD	0.648	0.650	0.686	0.744	0.699	0.625	0.640	0.700	0.703	0.724	0.627
Cronbach’s α	0.908	0.873	0.795	0.857	0.850	0.904	0.904	0.875	0.801	0.863	0.909
AVE	0.783	0.664	0.830	0.777	0.770	0.675	0.602	0.728	0.717	0.785	0.688
CR	0.935	0.908	0.907	0.913	0.909	0.926	0.923	0.914	0.884	0.916	0.930

*higher-order learning (HL), integrative learning (IL), and reflective learning (RL), knowledge-sharing (KS), problem-solving (PS), general ability for work (GAW), professional ability for work (PAW), attitude at work (AW), career planning and confidence (CPC), Career-decision Status (CDS). Italic values mean squared value of AVE.*

### Inner Model Analysis

To assess the structural model, Hair et al. (2017) suggested looking at the R^2^, beta (β) and the corresponding *t*-values *via* a bootstrapping procedure with a resample of 5,000. According to claims from Sullivan and Feinn (2012), “while a *p*-value can inform the reader whether an effect exists, it will not reveal the size of the effect. In reporting and interpreting studies, both the substantive significance (effect size) and statistical significance (*p*-value) are essential results to be reported (p. 279).” Before conducting hypotheses testing, this study must ensure that the values of the variance inflation factor (VIF) are less than 5, but the research results showed that the VIF values were between 1.377 and 2.274. Thus, there were no multicollinearity problems among the latent variables (Hair et al., 2017).

Figure 2 and Table 2 shows the results of the hypothesized relationships and standardized coefficients in inner model. The results showed that self-efficacy (β = 0.546, *p* < 0.001) and employability (β = 0.222, *p* < 0.001) were positively and significantly related to student career decision status, supporting H1 and H2. Similarly, self-efficacy (β = 0.207, *p* < 0.001) was positively and significantly related to student employability, supporting H3. In addition, our results found that deep approach to learning was positively and significantly related to self-efficacy (β = 0.494, *p* < 0.001) and employability (β = 0.265, *p* < 0.001), supporting H4 and H5. Finally, problem-based learning was positively and significantly related to self-efficacy (β = 0.287, *p* < 0.001) and employability (β = 0.318, *p* < 0.001), supporting H6 and H7. The Stone-Geisser Q2 values obtained through the blindfolding procedures for student employability (Q^2^ = 0.388), student self-efficacy (Q^2^ = 0.356) and student career decision status (Q^2^ = 0.336) were larger than zero, supporting the model has predictive relevance (Hair et al., 2017).

**FIGURE 1:**
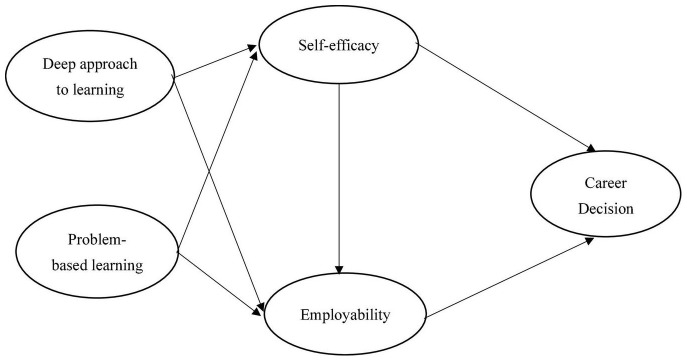
Research framework.

**FIGURE 2:**
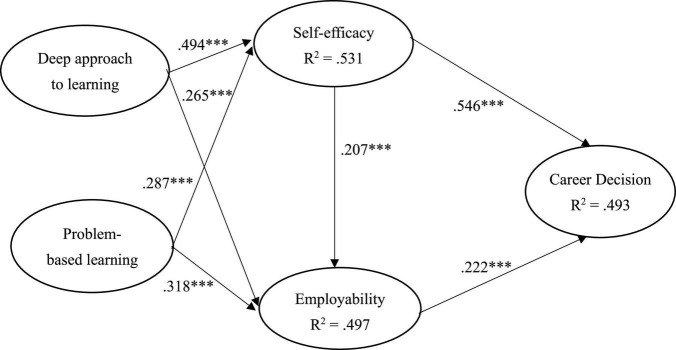
Results of structural model. ^***^ *if p* < 0.001.

**TABLE 2:** Results of the hypotheses testing.

Paths	β	error	*t*-value	Decision	Significance CI (2.50–97.5%)	VIF	*f* ^2^
H1: Employability → Career Decision	0.222	0.040	5.613	Support	CI (0.149–0.303)	1.555	0.062
H2: Self-efficacy → Career Decision	0.546	0.040	13.683	Support	CI (0.459–0.623)	1.555	0.379
H3: Self-efficacy → Employability	0.207	0.046	4.533	Support	CI (0.120–0.298)	2.133	0.040
H4: Deep learning → Self-efficacy	0.494	0.054	9.163	Support	CI (0.381–0.597)	2.086	0.250
H5: Deep learning → Employability	0.265	0.055	4.767	Support	CI (0.152–0.369)	2.606	0.053
H6: PBL → Self-efficacy	0.287	0.052	5.509	Support	CI (0.185–0.384)	2.086	0.084
H7: PBL → Employability	0.318	0.056	5.726	Support	CI (0.212–0.427)	2.261	0.089

*CI, Confidence intervals (Lower bound—Upper bound).*

### Examination of Mediating Effects

Student self-efficacy and student employability in the SOR model can be regarded as mediating variables. In order to verify whether both variables have mediating effects, a bootstrapping procedure is utilized to establish the structural model *via* Smart-PLS. Research results were shown in Table 3 indicated that indirect effects of student self-efficacy were significant, which supported H2a and H2b. It shows that mediating variables in the SOR model play important roles. Similar to the results of previous studies, student self-efficacy can enhance the effects of antecedents, forming strong psychological features, which are then reflected in socioeconomically disadvantaged students’ career decision status.

**TABLE 3:** Path coefficient of direct, indirect and total effects.

	Effect	Self-efficacy	Employability	Career decision status
Deep learning	Direct effect	0.494***	0.265***	- - - - -
	Indirect effect	- - - - -	0.102***	0.351***
	Total effect	0.494***	0.367***	0.351***
PBL	Direct effect	0.287***	0.318***	- - - - -
	Indirect effect	- - - - -	0.059**	0.241***
	Total effect	0.287***	0.377***	0.241***
Self-efficacy	Direct effect	- - - - -	0.207***	0.546***
	Indirect effect	- - - - -	- - - - -	0.046***
	Total effect	- - - - -	0.207***	0.592***
Employability	Direct effect	- - - - -	- - - - -	0.222***
	Indirect effect	- - - - -	- - - - -	- - - - -
	Total effect	- - - - -	- - - - -	0.222***

** if p < 0.01; *** if p < 0.001.

## Conclusion

### Discussions

Career decisions are among the most important decisions an individual makes in a lifetime, as they play a vital role in an individual’s social, economic, and emotional health. Such decisions are challenging for most people, and many find them stressful and can lead to indecision when it comes to career choices (Chuang et al., 2007; Gati and Kulcsár, 2021; Liu et al., 2022). Ultimately, the goal of educators is to prepare students for future careers. Belief that students benefit from the educational experience, mentoring, socializing, and their specific career development activities in the program (Chuang et al., 2007). Schools support and help students make career decisions and set realistic career expectations (Liu et al., 2022).

This research conducts SOR model to formulate a structural model that includes two learning methods for facilitating learning engagement, and explores how to enhance socioeconomically disadvantaged students’ career decision status from a process view. In the SOR model, although the interaction among stimulus, organism and response are emphasized, there is a foreseeable gap in the formation of the individual’s organism and its reflection in the subsequent behavior and attitude under the influence of external learning stimuli. The SOR model can help us more rigorously explain socioeconomically disadvantaged students’ psychological cognition and attitude development process stimulated by different learning methods, and the enhancement effect on their career decision status. The research results point out that the model has a good fit and has a positive and significant effect on all paths, which further strengthens the rationality of the model in this research.

The research results show that deep approach to learning and problem-based learning have positive and significant effects on self-efficacy and employability. In other words, socioeconomically disadvantaged students accept and adopt learning methods that are conducive to learning input in the learning process, can feel the confidence brought by learning and acquire valuable knowledge and skills, allow them to use the knowledge and skills to solve problems in adversity. The research findings show that the positive effects of deep approach to learning on self-efficacy and employability confirm to the research results from Burga et al. (2020), Dolmans et al. (2016), Peng and Chen (2019), Varunki et al. (2017), verified that the deep approach to learning helps students obtain more psychological and substantive results. In addition, different from the research of Vos et al. (2011), Peng and Chen (2019), this research uses socioeconomically disadvantaged students as a sample to better understand the contribution and role of socioeconomic status factors in student learning models, and explain how socioeconomically disadvantaged students can acquire the knowledge and skills they need through correct learning methods instead of recitation or mechanical learning methods.

Moreover, problem-based learning has attracted the attention of most scholars in the past literature and they have used various research methods to explore the effects of problem-based learning, but there are few research discussions on the impact of socioeconomically disadvantaged students. The research findings show that the positive effects of problem-based learning on self-efficacy and employability confirm to the research results from Martin et al. (2008), Liu et al. (2019), Liu et al. (2020), Peng et al. (2021), emphasizing that problem-based learning in socioeconomically disadvantaged students plays an important role in facing given tasks, and enrich the generality of the application in the SOR model. However, the majority of learners in typical problem-based learning studies are gifted or K12 and high school education students (Liu et al., 2019). There is a lack of research on problem-based learning use by socioeconomically disadvantaged students (Gallagher and Gallagher, 2013). Additionally, few research is found on problem-based learning use by students who are often from the disadvantaged and minority groups. This study aimed to fill this gap. Through problem-based learning, socioeconomically disadvantaged students can improve advanced thinking skills and perform well in a challenging problem-based environment.

Socioeconomically disadvantaged students’ positive feelings, attitudes and behaviors will be affected by the mutual links with effective learning methods. The research findings show that self-efficacy will positively affect student employability and career decision status. The research results are similar to those from Liu et al. (2020), Xu et al. (2021) and Zhao et al. (2021) that is, high self-efficacy can make students accessible to acquiring employability and improve career decision status in a more effective way. The research results support this argument, and self-efficacy’s role as a mediator in the SOR model has also been verified. These results are similar to previous studies (Liu et al., 2020; Xu et al., 2021). When socioeconomically disadvantaged students realize that they have the ability to solve problems and accomplish the goals they want to achieve, the self-confidence they gain through effective learning methods (such as deep approach to learning and problem-based learning) can increase their mental energy and the knowledge and skills needed in studying for employment, and to further improve their choice of future career orientation. In addition, through the verification of mediating effect, the results of this study confirm that self-efficacy and employability have an intermediary effect in the SOR model, which means that socioeconomically disadvantaged students who enter the learning context setting of deep approach to learning and problem-based learning can not only improve the confidence in completing goals and tasks, but also improve the understanding of employment knowledge and skills, from the familiarity of self-confidence and skills to a clearer grasp of future career decision status.

### Educational Implications

Practically, the results of this study may provide useful guidance to higher education institutions, faculties and teachers on career decision and development of socioeconomically disadvantaged students. The research results point out that deep approach to learning and problem-based learning has a significant positive impact on self-efficacy and employability, It means that the higher the degree of investment of socioeconomically disadvantaged students in these two learning situations, the more they can increase their self-confidence in accomplishing their goals and the knowledge and skills needed for employment. Because socioeconomically disadvantaged students have a long-term lack of resources, they have a clear understanding of the academic support they need. Compared with other socioeconomically advantaged students, they know how to learn to use fewer resources to achieve similar results and solve problems and challenges. This research suggests that schools should provide socioeconomically disadvantaged students with more work-study opportunities for social services and internships, and mentors should guide students to engage in social services and corporate internships, and replace surface or traditional learning in the classroom through the internship process.

The findings state that students with high self-efficacy and employability will increase their degree of career decision status, means that socioeconomically disadvantaged students recognize that their psychological state and employment are stronger, and they will be more autonomous in their future career decision status. This research suggests that schools should provide substantial employment counseling services for socioeconomically disadvantaged students, and use scientific test design to confirm students’ personality quality, career orientation and employment skills, and provide students with reference for career choices and decisions, so that students can better understand own core competitiveness.

### Research Limitations and Directions for Future Studies

The research findings make a contribution to the literature on SOR model and student career development. However, some limitations still exist and reveal directions for further research. First, despite there is considerable status in the field of psychological for the SOR model, the relationship between learning ways and career decision status of socioeconomically disadvantaged students engaged in higher education is taken into consideration by only a few studies. Notwithstanding that SOR model was taken as reference to establish deep approach to learning and problem-based learning in the study, and significant learning theories can be acquired from the research findings, other motivation theories, including theories of attribution, self-efficacy, and hierarchy needs, are still utilized to account for how to stimulate learning in students who are socioeconomically disadvantaged. Therefore, subsequent research is suggested to make use of different theoretical models to figure out related psychological dimensions which influence career decision status. Second, students were required to give self-report details on their learning method as the indicator, of which the main reason lies in the confidentiality of actual data which is not easily accessible. However, in the self-statement of psychological status from students, errors may occur. Considering research ethics, if the actual psychological status of students is assessed, it may be easier to understand the connection between learning methods and career decision status. In addition, subsequent researchers are suggested to bring interview contents and students’ observations of learning status to their studies, so as to provide support for the research findings and give an overall judgment. Third, only six universities in Taiwan were taken as samples in the study and 627 valid copies of questionnaire in total were collected on account of time and space restrictions. In addition to enlarging the quantity of samples and improving the representativeness of research, other groups or regions could be compared and explored in subsequent research, so that additional insights related to policies of higher education can be available. Ultimately, Wu (2020) argued that differences may exist in after-school and in-class psychological cognitive results derived from students, and an unsolved black box also occurs between them. Nevertheless, there was no analysis for this classification in this study. Thereby, it is suggested in the study that researchers need to make a comparison of them and provide more valuable insights into the unsolved black box.

## Data Availability Statement

The raw data supporting the conclusions of this article will be made available by the authors, without undue reservation.

## Ethics Statement

The studies involving human participants were reviewed and approved by Academic Committee of Business School, Foshan University. The patients/participants provided their written informed consent to participate in this study.

## Author Contributions

MP and XY contributed to the conceptual framework building, methodology, and discussion. Both authors contributed to the article and approved the submitted version.

## Conflict of Interest

The authors declare that the research was conducted in the absence of any commercial or financial relationships that could be construed as a potential conflict of interest.

## Publisher’s Note

All claims expressed in this article are solely those of the authors and do not necessarily represent those of their affiliated organizations, or those of the publisher, the editors and the reviewers. Any product that may be evaluated in this article, or claim that may be made by its manufacturer, is not guaranteed or endorsed by the publisher.
